# Data mining polycystic ovary morphology in electronic medical record ultrasound reports

**DOI:** 10.1186/s40738-019-0067-7

**Published:** 2019-12-01

**Authors:** Jay Jojo Cheng, Shruthi Mahalingaiah

**Affiliations:** 1 Department of Biostatistics and Medical Informatics, University of Wisconsin, 702 West Johnson Street, Madison, WI 53792 USA; 2 Department of Obstetrics and Gynecology, Boston University School of Medicine, 85 East Concord Street, Boston, MA 02118 USA; 3 Department of Epidemiology, Boston University School of Public Health, Talbot 3E, 715 Albany Street, Boston, MA 02118 USA; 4 Department of Physiology & Biophysics, Boston University School of Medicine, 700 Albany St. W302, Boston, MA 02118 USA

**Keywords:** Machine learning, Data mining, Ultrasound, Polycystic ovary syndrome, Electronic medical record

## Abstract

**Background:**

Polycystic ovary syndrome (PCOS) is characterized by hyperandrogenemia, oligo-anovulation, and numerous ovarian cysts. Hospital electronic medical records provide an avenue for investigating polycystic ovary morphology commonly seen in PCOS at a large scale. The purpose of this study was to develop and evaluate the performance of two machine learning text algorithms, for classification of polycystic ovary morphology (PCOM) in pelvic ultrasounds.

**Methods:**

Pelvic ultrasound reports from patients at Boston Medical Center between October 1, 2003 and December 12, 2016 were included for analysis, which resulted in 39,093 ultrasound reports from 25,535 unique women. Following the 2003 Rotterdam Consensus Criteria for polycystic ovary syndrome, 2000 randomly selected ultrasounds were expert labeled for PCOM status as present, absent, or unidentifiable (not able to be determined from text alone). An ovary was marked as having PCOM if there was mention of numerous peripheral follicles or if the volume was greater than 10 ml in the absence of a dominant follicle or other confounding pathology. Half of the labeled data was used to develop and refine the algorithms, and the other half was used as a test set for evaluating its accuracy.

**Results:**

On the evaluation set of 1000 random US reports, the accuracy of the classifiers were 97.6% (95% CI: 96.5, 98.5%) and 96.1% (94.7, 97.2%). Both models were more adept at identifying PCOM-absent ultrasounds than either PCOM-unidentifiable or PCOM-present ultrasounds. The two classifiers estimated prevalence of PCOM within the whole set of 39,093 ultrasounds to be 44% PCOM-absent, 32% PCOM-unidentifiable, and 24% PCOM-present.

**Conclusions:**

Although accuracy measured on the test set and inter-rater agreement between the two classifiers (Cohen’s Kappa = 0.988) was high, a major limitation of our approach is that it uses the ultrasound report text as a proxy and does not directly count follicles from the ultrasound images themselves.

## Background

Polycystic ovary syndrome (PCOS) is a leading cause of female infertility and one of the most common endocrine disorders in women [1]. Affecting about 5–15% of reproductive-age women, the syndrome is characterized by hyperandrogenism, oligo-anovulation, and polycystic ovary morphology (PCOM), and it is associated with an increased risk for infertility, endometrial cancer, and metabolic syndrome [2]. Despite its prevalence, the syndrome’s etiology is not well understood and its diagnosis is contested [3].

PCOM, in particular, plays a central role in the ongoing deliberation: the two leading criteria, the 2003 Rotterdam Consensus Criteria and the Androgen Excess and PCOS Society recommendations, diverge primarily on the sufficiency of PCOM as a criterion in the diagnosis of PCOS. There is recent evidence that the improved resolution of newer ultrasound technology increases the number of observable follicles, thereby inflating the prevalence of PCOM [4]. Furthermore, PCOM is a relatively common finding in healthy women with robust early follicular recruitment [5, 6]. For these reasons, some propose increasing the follicle count cutoff or abandoning ultrasound altogether in favor of other biomarkers, such as serum anti-Müllerian hormone (AMH) [7, 8].

The role of PCOM within the disease is difficult to study in part because of the blood tests, pelvic ultrasounds, and accurate menstrual data required for studies on PCOS. Existing electronic medical record (EMR) data provide an opportunity for a closer study of PCOM and suggest appropriate diagnostic standards. Compared to the high costs associated with collecting and interpreting new ultrasound data, hospital data is abundant, readily available, relatively inexpensive, and linkable with patients’ other clinical data for longitudinal investigation. The major challenge with using EMR data is that information about PCOM is captured in formats not amenable to traditional methods, such as ultrasound images and radiology text reports.

In this study, we develop two text-based machine learning algorithms for identifying PCOM in pelvic ultrasound reports and compare them to human benchmarks. These tools work directly with text from the report, leveraging the fact that even if a clinician may not be actively reporting about PCOM in writing the ultrasound report, its presence or absence can be inferred through reported volume measurements and what is written about the ovaries’ internal structure.

## Methods

### Data and study design

All ultrasounds from October 1, 2003 to December 7, 2016 were queried from the Boston Medical Center Clinical Data Warehouse. The start-date was selected to reflect the first day that ICD-9 codes were used and recorded at Boston Medical Center. This query yielded 39,093 ultrasounds for 25,535 unique patients; in total, this text corpus consisted of 3,707,837 words, with each ultrasound document containing an average of 95 words. Using a regular expression search, a standard practice for string-searching algorithms, we found that out of 39,093 ultrasounds, only 6273 did not include three-dimensional volume measurements of the ovaries. These ultrasounds were still included for classification. This study was approved by the Institutional Review Board of Boston University School of Medicine and the Boston University Medical Campus.

### Reference label definition

Of the 39,093 ultrasounds, 2000 reports were randomly selected to be hand-labeled by research staff supervised by a board-certified reproductive endocrinology and infertility (REI) specialist. These labels served as the human benchmarks for the study. Of these, 1000 randomly selected ultrasounds were chosen to be a training set for developing the algorithms and another 1000 randomly selected ultrasounds were chosen to be a test set for an unbiased estimate of its performance.

The 2003 Rotterdam Criteria define PCOM as the “presence of 12 or more follicles in each ovary measuring 2-9 mm in diameter, and/or increased ovarian volume (10 mL).” [2] Following the Criteria, an ovary was labeled with one of three determinations: polycystic morphology (PCOM-present), free of polycystic morphology (PCOM-absent), or unidentifiable in the current examination (unidentifiable). We classified an ovary as PCOM if it was greater than 10 ml in volume in the absence of a dominant follicle (or other confounding structure), or if it contained a mention of classical PCOM appearance, such as “string of pearls orientation,” or “numerous peripheral follicles.” A determination of unidentifiable was made if the volume was greater than 10 ml, but there was also mention of a structure that confounded stromal volume (e.g. dominant follicle, dermoid cyst, hemorrhagic cyst, etc.), if there was no mention of ovaries, or if the ovaries were not measured. This follows the Rotterdam consensus that “If there is evidence of a dominant follicle (>10mm) or a corpus luteum, the scan should be repeated the next cycle.” An ovary was considered PCOM-absent if it was smaller than or equal to 10 ml and did not describe classical PCOM appearance.

### Models

**Fig. 1:**
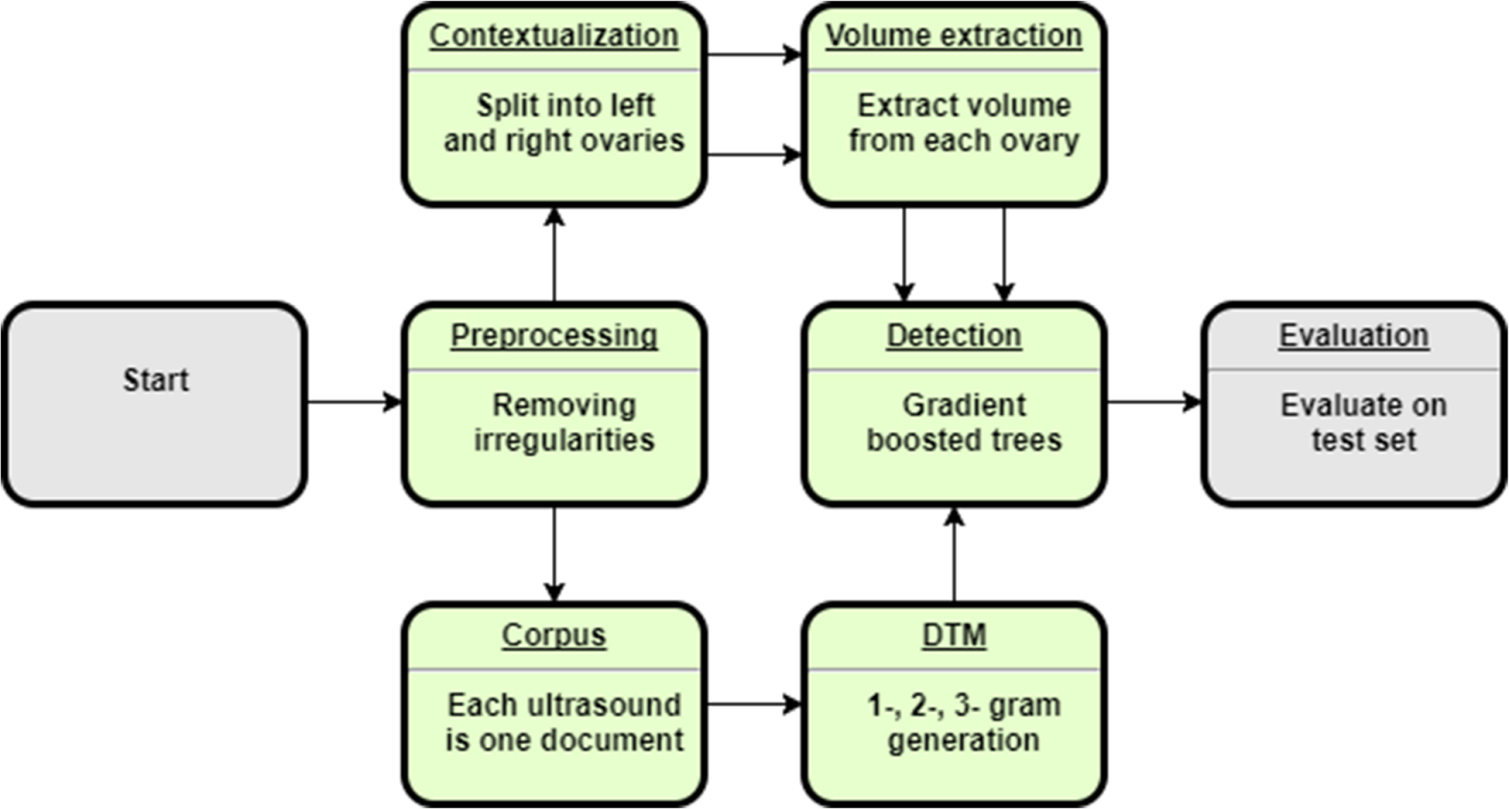
Schematic for the Gradient Boosted Tree PCOS Text Classifier

**Fig. 2:**
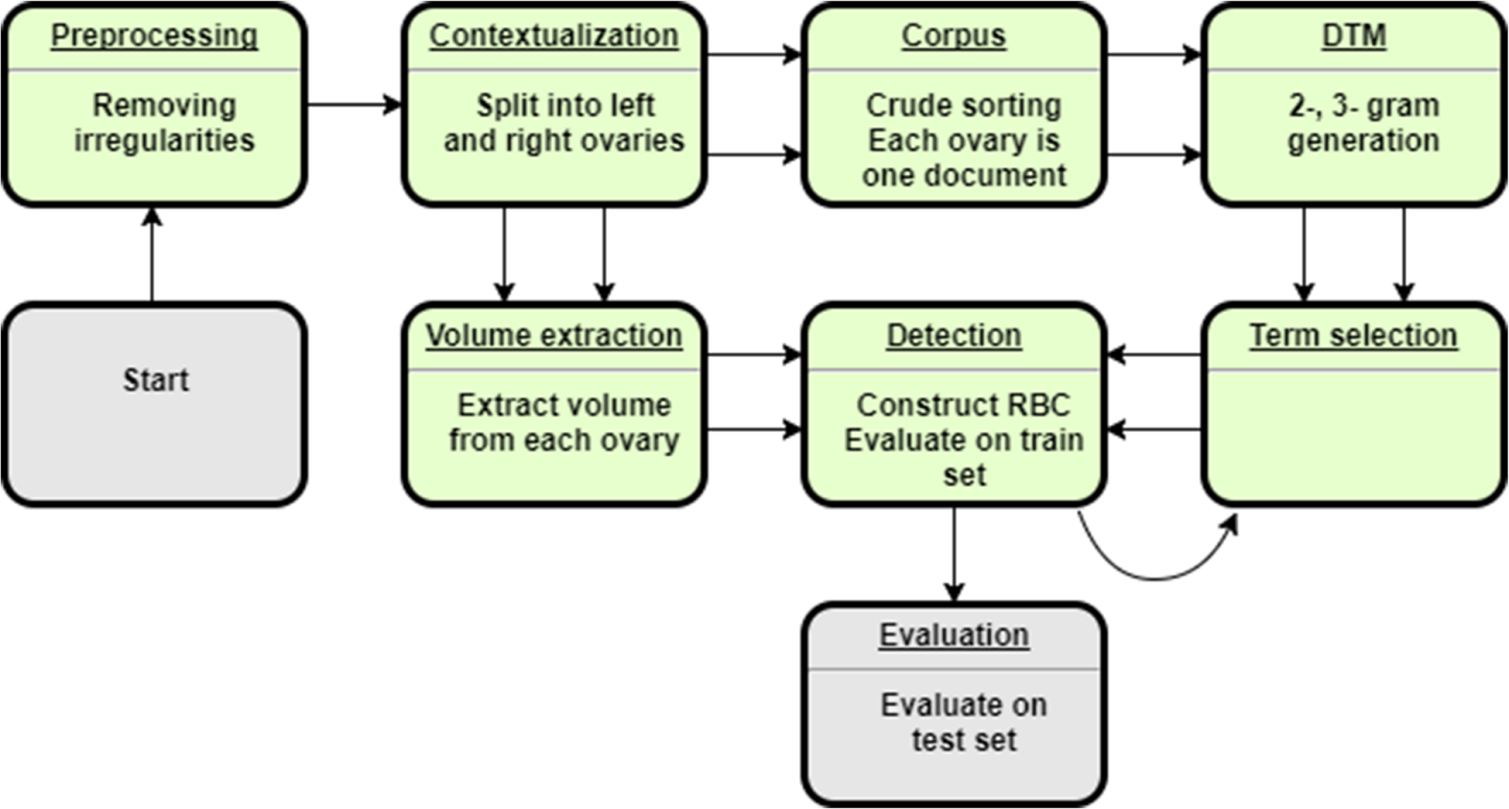
Schematic for the Rules-Based PCOS Text Classifier

To accomplish the classification task, two approaches were used: (1) a rule-based classifier (RBC) that checks the size of each ovary and detects important words appearing close to the word, “ovary,” which may indicate the presence of PCOM or confounding structures, and (2) a gradient boosted tree model (GBT) that learns from the frequencies of key terms and ovarian volume measurements within each report. Since neither algorithm uses the image directly and antral follicle counts not always reported directly, follicle counts are not used. The overall architectures are depicted in Figs. 1 and 2, respectively.

### Rule-based classifier (RBC)

The rule-based classifier consists of several modules: a preprocessing phase, contextualization, volume extraction, corpus and document-term matrix creation, term selection, detection, and final evaluation. The overall scheme is given in Fig. 1.

### Text preprocessing

In the preprocessing phase, standard NLP techniques for cleaning are used, including removing irregular characters, nonstandard spaces, punctuation, and stopwords (words like ‘and’ or ‘the’). Each word is then stemmed (trimmed to a root) with Porter’s Snowball algorithm. This phase ensures that these irregularities do not affect the analysis and that different forms of a word are counted as the same word.

### Contextualization, volume extraction, corpus creation

In the contextualization module, the scope of text used for prediction is limited. Only the sentences mentioning the word “ovary,” as well as the sentences immediately succeeding such sentences are extracted to discard descriptions of other organs such as the uterus or kidneys, which may also appear on pelvic ultrasounds. Next, regular expressions are used to group sentences with either the left or right ovary. The end-product of this module is a dataset with up to two entries corresponding to a single ultrasound (one for either ovary). Sentences that describe both ovaries are repeated and appear in both rows. The group of sentences associated with a single ovary are henceforth referred to as a ‘document,’ and the collection of these documents is the whole ‘corpus.’

During the volume extraction phase, regular expressions are used to extract 3-dimensional measurements from the ultrasounds and to determine the correct unit of measurement. Volume of ovaries are calculated according to $length\cdotp width\cdotp height\cdotp \frac{\pi }{6}$ [9]. Ovarian measurements with only 1- and 2-dimensions reported are excluded (as a volume cannot be computed).

### Document-term-matrix (DTM) creation

In order to use the volume cutoff of 10 ml, we needed to distinguish between ovaries enlarged by increased stromal volume (PCOM) and ovaries enlarged due to recruitment of a dominant follicle or presence of abnormal pathology (e.g. hemorrhagic cysts and dermoids). The text that indicated the latter circumstance are called ‘volume confounders.’ We constructed a comprehensive list of reasons empirically using a document-term matrix (DTM). This matrix is essentially a frequency table of words in the documents. The columns of the matrix correspond to every word that appears at least once in the corpus (in at least one document), and the rows correspond to each document. The cells correspond to the number of times the term is used in the document. Using the DTM offers the distinct advantage of generating all possible reasons appearing in the dataset, implicitly accounting for the natural language of the data. Typically the problem of listing these reasons would be impossible to solve a priori.

With the volumes extracted previously, the algorithm finds 2-, and 3-g (2 and 3 word groupings) correlated with a large ovarian volume (> 10 ml) were extracted into a list [10]. We chose not to use 1-g in order to decrease the variance of the method. The list of 3-g correlated to “numerous peripheral follicles” and the list of 2-g most correlated with the phrases, “ovarian syndrome,” “polycystic ovarian,” and “string of pearls” were also extracted into a list in order to detect the noted presence of PCOM. This module results in two lists of n-grams: one for potential volume confounders and the other for potential descriptions of PCOM.

### Term selection and detection

The n-grams from these two lists were then curated so only phrases that indicate the presence of polycystic morphology or the presence of a volume confounder were kept (Additional file 1: Tables S1 & 2). During the detection phase, the relative counts of these phrases were used to determine either the presence of volume confounding or polycystic morphology. This selection of n-grams was iteratively refined on the training set.

The rules for the detection algorithm then followed the Rotterdam Consensus Criteria and used the ovarian volume and presence of numerous follicles to determine the presence of PCOM for each ovary (Additional file 1: Figure S3). Overall patient status for the given ultrasound is determined from the status of both ovaries. The patient is determined to have PCOM on a given ultrasound if at least one ovary had PCOM.

### Evaluation

During the evaluation phase, predicted classification labels were compared to the hand-labeled test set of 1000 ultrasounds for computing confusion matrices and accuracy statistics.

### Gradient boosted tree classifier (GBT)

Gradient boosting is a machine learning technique that ensembles many weak prediction models in an additive, stage-wise manner to produce a stronger model. At each stage, new weak models are introduced to account for the errors of previously existing weak models, allowing the ensemble to learn non-linearities [11, 12]. The schematic for gradient boosted tree model is given in Fig. 2. The GBT uses the same preprocessing module as the RBC. After the ultrasound text is preprocessed, the corpus is generated directly without contextualization and splitting into left and right ovaries. Thus, each ultrasound report corresponds to one document within the corpus. This was done to see if the bilateral nature of detecting PCOM could be learned by the algorithm without explicitly programming it. Similarly, a document-term matrix was generated from all 1-, 2-, and 3-g without any human selection to see if important phrases could be learned during training.

The contextualization and volume extraction modules were used to extract ovarian volumes as predictors for the boosted tree algorithm. The GBT used the XGBoost library with default parameters except for the number of training iterations, which was selected by minimizing the 5-fold cross-validation (CV) error on the training data [13]. Hyperparameter tuning was briefly attempted, but abandoned because there were no significant improvements in CV training error compared to the extra computational cost. Additional file 1: Figure S4 shows the most important predictors learned by the GBT model.

## Results

Table 1 includes demographic and general health information about the source population at Boston Medical Center. Compared to other cohorts, this group has substantial race/ethnic and socio-economic diversity. Nearly 80% of the individuals are non-white, with a third of the patients not finishing high school, another third attaining high school diplomas or GED, and a third with at least some higher education. Nearly 2% have been homeless, compared to 0.5% of the United States population [14]. The diversity of the cohort reflects the diverse patient population served by Boston Medical Center, which serves as the region’s safety net hospital. Blood pressure is within a normal range and about a third of the patients are underweight or normal, another third are overweight, and a third are obese.

**Table 1:** Source population characteristics

*N* = 25,535	% (n) or mean (SD)
Age	
Age at ultrasound	31.4 (7.9)
Race	
Asian	3.5% (883)
Black/African American	48.9% (12,489)
Hispanic/Latino	19.9% (5076)
Non-Hispanic White	21.0% (5358)
Other*/Multiracial	5.6% (1432)
Declined/Not Available	1.2% (297)
Vitals	
BMI (*n* = 18,443)	
Underweight < 18.5	1.2% (225)
Normal weight 18.5–24.9	27.9% (5138)
Overweight 25–29.9	30.4% (5610)
Obese Class I 30–35	20.7% (3818)
Obese Class II 35–40	11.2% (2060)
Obese Class III > 40	8.6% (1592)
SBP (mmHg) (*n* = 21,353)	118.8 (11.6)
DBP (mmHg) (*n* = 21,351)	75.2 (7.6)
Age at Menarche (*n* = 178)	12.7 (2.0)
Socioeconomic Status	
Education (*n* = 39,638)	
Did not attend school	5.6% (1416)
8th grade or less	4.6% (1169)
Some high school	22.3% (5690)
Graduated high school or GED	27.1% (6928)
Some college/vocational/technical school	13.4% (3426)
Graduated college/postgrad.	16.8% (4292)
Other Education	1.0% (266)
Declined/Not Available	9.2% (2348)
Ever homeless	
Yes	1.9% (486)
No	98.1% (25,040)
Not available	< 0.1% (9)

*Includes groups with less than 250 members: American Indian/Native American, Middle Eastern, and Native Hawaiian/Pacific Islander

*SBP* Systolic Blood Pressure; *DBP* Diastolic Blood Pressure

Table 2 presents the test set performance of the two models. The overall test set accuracy of the RBC was 97.6% (95% CI: 96.5, 98.5%) and 96.1% (94.7, 97.2%) for the GBT. The RBC slightly outperforms the gradient boosted trees in correctly classifying ultrasounds as unidentifiable and PCOM-present, but the GBT would scale to other datasets more readily. Both models were more adept at identifying PCOM-absent ultrasounds than either unidentifiable or PCOM-present ultrasounds. Table 3 presents classification frequencies predicted by the two classifiers for the entire dataset and their confusion matrix. Inter-rater agreement between the two classifiers was high (Cohen’s Kappa = 0.988) and the two classifiers estimated prevalence of PCOM within our population’s ultrasounds to be about 44% PCOM-absent, unidentifiable 32, and 24% PCOM-present.

**Table 2:** Classification metrics across different models

Model	Accuracy	Class label	Sensitivity	Specificity	PPV	NPV
Gradient boosted trees	0.961 (0.9471, 0.9721)	PCOM-absent	0.9977	0.9982	0.9977	0.9982
		Unidentifiable	0.9474	0.9705	0.9387	0.9748
		PCOM-present	0.9146	0.9761	0.9259	0.9723
Rules-based classifier	0.976 (0.9645, 0.9846)	PCOM-absent	0.9885	0.9982	0.9977	0.9912
		Unidentifiable	0.966	0.9852	0.9690	0.9838
		PCOM-present	0.9668	0.9829	0.9472	0.9894

*PPV* Positive predictive value; *NPV* Negative predictive value

**Table 3:** Classification frequencies by model and confusion matrix

Rules-based classifier					
		PCOM-absent	Unidentifiable	PCOM-present	
Gradient boosted trees	PCOM-absent	17,371	5	7	44.5% (17,383)
	Unidentifiable	0	12,311	111	31.8% (12,422)
	PCOM-present	3	175	9110	23.8% (9288)
		44.4% (17,374)	32.0% (12,491)	23.6% (9228)	Kappa: 0.9881

*PCOM* Polycystic ovary morphology

## Discussion

### Major findings

We have developed two methods for determining the presence of polycystic ovarian morphology in ultrasound reports that demonstrate high sensitivity and specificity with the rules-based classifier performing slightly better than the gradient-boosted trees (97.6% vs 94.7% accuracy). The two algorithms similarly predict the prevalence of PCOM in our dataset to be 24%, which is comparable to the 22% reported by Clayton et al. in unselected women [5], and 23% reported by Polson et al. in women who considered themselves healthy [15]. Our intuition is that both methods likely work well within the current domain because the highly sequential nature of the Rotterdam Consensus Criteria is amenable to decision trees.

The RBC was successful because the classification of ultrasounds could be reduced to considering a relatively small set of 600 n-grams (Additional file 1: Tables S1 & 2). This approach may generalize well to applications where a disease in question is associated with highly specific language and generalize more poorly to identifying pathologies that overlap with others in symptomatology. Another advantage is that this method requires little training data for development– thus, for a given labeled dataset, more of the data can be apportioned to estimating its accuracy.

The GBT classifier holds the advantage of a faster development time and better portability to other datasets. Compared to the RBC, domain expertise is less critical and performance increases with additional data. The GBT’s non-deterministic nature also allows it to generate probabilities for the other classes, giving its predictions a measure of uncertainty which can then be propagated within hierarchical Bayesian models which incorporate its predictions, making it a viable choice for incorporating into larger analytical projects.

### Error analysis and limitations

Analysis of incorrect predictions reveals that the RBC model has difficulty with temporal language. For example, it may detect that a radiologist is writing about a large cyst, but it does not recognize that this cyst existed in the past and is no longer seen in the current exam. Both models have difficulty classifying ultrasounds when the ovarian volume is not reliably extracted, usually due to typographical errors arising from dictation software. Nevertheless, both models were surprisingly error tolerant, because often classifying a single ovary correctly was enough to classify the overall status.

Other errors of the GBT model seemed to arise from the limitations of not utilizing word order, so it had difficulty detecting long-range interactions. For example, the text for one ultrasound reads, “the right ovary…containing 3.5x4 cm hemorrhagic cyst. the left ovary measures 3x3x5 cm.” The RBC would have split these two sentences and assigned them to each ovary, but the GBT considers the entire set of n-grams, which captures the three words, “cyst. left ovary” as a single unit, ultimately guessing that a cyst is present in the left ovary since the word “cyst” is closer in position to the phrase “left ovary.”

When predictions for the rest of the full dataset were generated by the two methods, the two classifiers agree strongly on what does not count as PCOM. Most of the disagreement is on a subset of 286 cases that seem to be either PCOM-present or unidentifiable. Out of the 1000 ultrasounds in the test set, there were 16 cases that were misclassified by both. We suspect that the test performance of the RBC model is slightly misleading as the test set accuracy is higher than the training set accuracy. A more conservative estimate of accuracy measured on the combined training and test set is 0.968. The optimistic assessment of performance seems to stem from the test set containing examples that were “easier” for the RBC to classify by random chance.

A general limitation of the approach outlined here is that inferring PCOM status from ultrasound reports can be inexact, because a radiologist may not explicitly describe everything that appears in the image, only aspects of the ultrasound relevant to the indication. Thus, the absence of an object within a report does not guarantee its absence in the image. However, methods that work directly with image data require that the images are properly labeled, and the tools presented here can be used to generate such labels.

## Conclusions

Current open questions about PCOM include estimating the within-woman variability of ovarian volume measurements over time and understanding race-ethnic characteristics of follicle counts. Historical ultrasound data collected as part of natural clinical processes could be a source of answers to these inquiries, but methods for harnessing this data are lacking. Here we developed two NLP classifiers that provide a means of addressing these questions. There is strong agreement between the two methods (Kappa = 0.988). The Rules-Based Classifier predicts 44.4% (17,374) PCOM-absent, 32.0% (12,491) unidentifiable, and 23.6% (9228) PCOM-present and the Gradient Boosted Tree model predicts 44.5% (17,383) PCOM-absent, 31.8% (12,422) unidentifiable, and 23.8% (9288) PCOM-present. These results are consistent to the approximately 20% prevalence of PCOM in some studies. We recommend that further research in automatic feature extraction for PCOS be directed towards methods for the raw ultrasound images themselves.

## Supplementary information

**Additional file 1:** **Table S1.** Confounder - These are phrases that indicate the presence of a volume confounder (words that indicate large volume is due to something other than PCOS). **Table S2.** PCOS words - These are phrases that indicate presence of polycystic morphology. **Figure S3.** Pseudocode - This is the pseudocode for the rules-based classifier routine. It implements the Rotterdam Consensus Criteria. **Figure S4.** Importance plot - The variables (word stems) determined to be most important for classification using the gradient boosted tree classifier. The top two variables, eval_right and eval_left are variables about the left and right ovarian volume extracted from the text.

## Data Availability

The datasets used and/or analyzed during the current study are available from the corresponding author on reasonable request.

## Abbreviations

AMH: Anti-Müllerian hormone
CV: Cross-validation
DTM: Document-term matrix
EMR: Electronic medical record
GBT: Gradient boosted tree model
PCOM: Polycystic ovary morphology
PCOM-absent: Free of polycystic morphology
PCOS: Polycystic ovary syndrome
RBC: Rule-based classifier
REI: Reproductive endocrinology and infertility
Unidentifiable: Unidentifiable in the current examination
