# Supplementary material for: Data mining polycystic ovary morphology in electronic medical record ultrasound reports
Source: Fertil Res Pract. 2019 Dec 1;5:13. doi: 10.1186/s40738-019-0067-7 (PMC6886196; doi:10.1186/s40738-019-0067-7)
Supplement: Supplementary file 1 — Additional file 1: Table S1. Confounder - These are phrases that indicate the presence of a volume confounder (words that indicate large volume is due to something other than PCOS). Table S2. PCOS words - These are phrases that indicate presence of polycystic morphology. Figure S3. Pseudocode - This is the pseudocode for the rules-based classifier routine. It implements the Rotterdam Consensus Criteria. Figure S4. Importance plot - The variables (word stems) determined to be most important for classification using the gradient boosted tree classifier. The top two variables, eval_right and eval_left are variables about the left and right ovarian volume extracted from the text. [file 40738_2019_67_MOESM1_ESM.zip › S3 pseudocode.pdf]

Algorithm 1 Rules-based detection pseudocode

```
procedure Calculate ovarian volumes
    for all ovaries do
        extract ovarian measurements with regular expressions
procedure Determine presence of volume confounders
procedure Determine presence of numerous peripheral follicles
procedure Classifying each ovary
    for all ovaries do
        if this ovary has “numerous peripheral follicles” then
            mark it as PCOM-present
        else if this ovary is larger than 10 ml then
            check for the presence of a volume confounder.
            if a ”volume confounder” is present in this ovary then
                mark the ovary as “undiscernible in this ultrasound”
            else
                mark the ovary as PCOM-present
        else if this ovary is smaller than 10 ml then
            mark the ovary as PCOM-absent
        else if this ovary is not visualized then
            mark the ovary as “undiscernible in this ultrasound”
procedure Classifying patient status
    for all patients do
        if either ovary is PCOM-present then
            the subject has PCOM
        else if both ovaries are PCOM-absent then
            the subject does not have PCOM
        else if one ovary is PCOM-absent and the other is undiscernible then
            the subject does not have PCOM
        else if both ovaries are undiscernible then
            the subject is undiscernible in this evaluation
```
