# Supplementary figures and images for: Data mining polycystic ovary morphology in electronic medical record ultrasound reports
Source: Fertil Res Pract. 2019 Dec 1;5:13. doi: 10.1186/s40738-019-0067-7 (PMC6886196; doi:10.1186/s40738-019-0067-7)

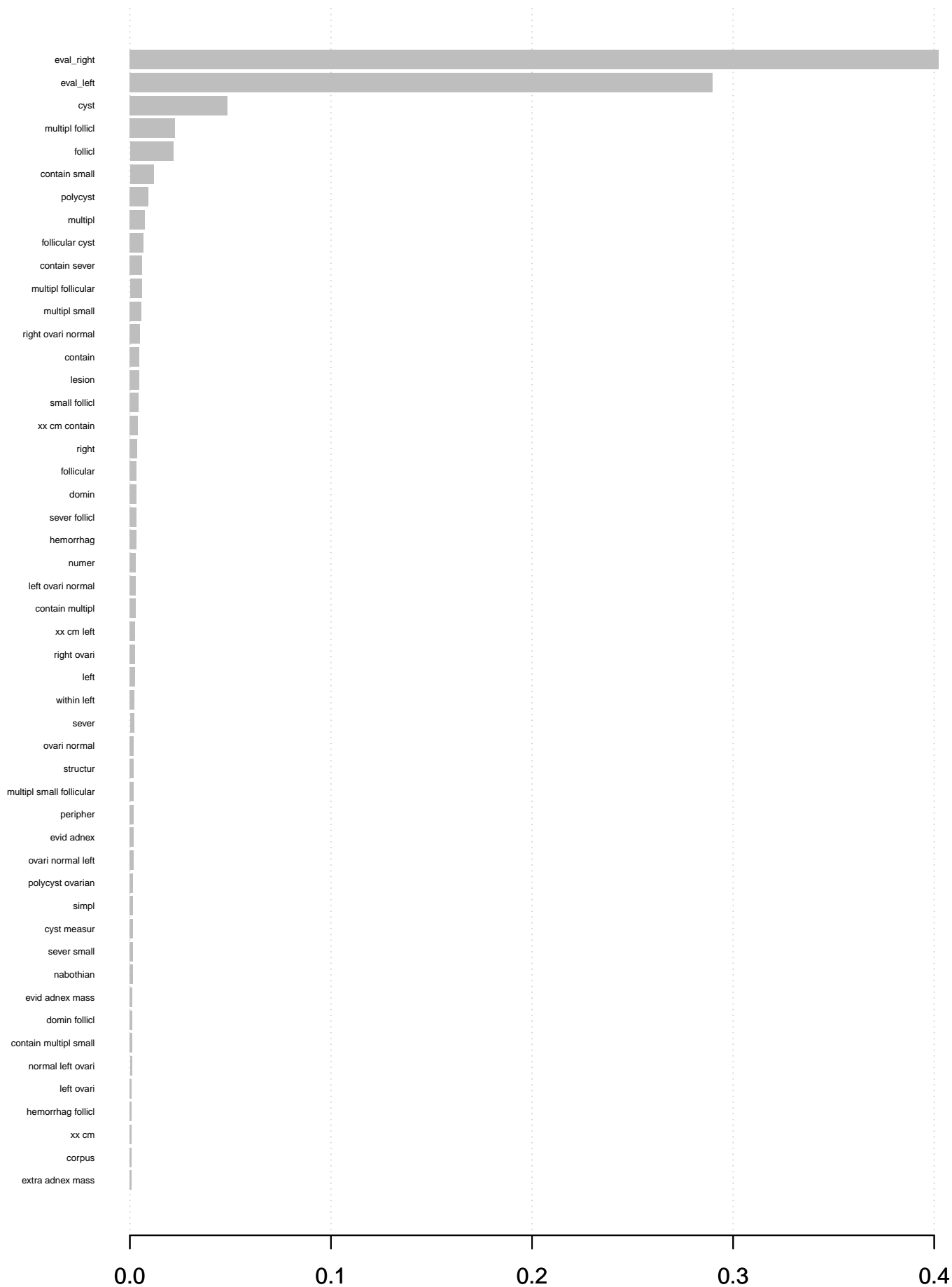

Supplement: Supplementary file 1 — Additional file 1: Table S1. Confounder - These are phrases that indicate the presence of a volume confounder (words that indicate large volume is due to something other than PCOS). Table S2. PCOS words - These are phrases that indicate presence of polycystic morphology. Figure S3. Pseudocode - This is the pseudocode for the rules-based classifier routine. It implements the Rotterdam Consensus Criteria. Figure S4. Importance plot - The variables (word stems) determined to be most important for classification using the gradient boosted tree classifier. The top two variables, eval_right and eval_left are variables about the left and right ovarian volume extracted from the text. [file 40738_2019_67_MOESM1_ESM.zip › S4 importance plot.pdf]
